# Impact of sperm chromatin damage on natural fertility and ART success: evidence- based medicine

**DOI:** 10.1186/1471-2164-15-S2-O24

**Published:** 2014-04-02

**Authors:** Ashok Agarwal

**Affiliations:** 1 Center for Reproductive Medicine, Glickman Urological and Kidney Institute, Cleveland Clinic and the Case Western Reserve University, Cleveland, Ohio, USA

## 

Diagnosis of male infertility has been based mainly on the traditional semen parameters (concentration, motility and morphology). Historically, the semen analysis results are the foundation on which clinicians base their decision for treatment for a given couple. It has, however, become clear that semen parameters are insufficient for the determination of male fertility potential. A continuous search for better markers of male fertility has led to an increased focus on testing of sperm chromatin integrity in fertility workup and ART. Sperm DNA damage is a useful biomarker for male infertility diagnosis and prediction of assisted reproduction outcomes. It is associated with reduced fertilization rates, embryo quality and pregnancy rates, and higher rates of spontaneous miscarriage and childhood diseases. Successful fertilization of the human oocyte from spermatozoa with damaged DNA may lead to paternal transmission of defective genetic material with adverse consequences to embryo development. Sperm DNA fragmentation has shown to be an independent predictor of success in couples undergoing intrauterine insemination (IUI). The speaker will discuss the conflicting reports on the role of sperm DNA fragmentation in relation to fertilization, pre-embryo development and pregnancy outcome in *in vitro* fertilization (IVF) and intra-cytoplasmic sperm injection (ICSI).

The speaker will provide a summary of the most recent studies in the literature in the field of sperm DNA damage in the clinical setting. He will discuss current laboratory tests and the accumulating body of knowledge concerning the relationship between sperm DNA damage and clinical outcomes. Next he will talk about the pros and cons and clinical applicability of the current sperm DNA fragmentation assays and the biological significance of sperm chromatin damage in the male germ line. Finally, as sperm DNA damage is often the result of increased oxidative stress in the male reproductive tract, the potential contribution of antioxidant therapy in the clinical management of this condition will be debated.

